# Feasibility of a Telerehabilitation Adaptation of the *Be Clear* Speech Treatment Program for Non-Progressive Dysarthria

**DOI:** 10.3390/brainsci12020197

**Published:** 2022-01-31

**Authors:** Brooke-Mai Whelan, Deborah Theodoros, Louise Cahill, Atiyeh Vaezipour, Adam P. Vogel, Emma Finch, Anna Farrell, Elizabeth Cardell

**Affiliations:** 1 Recover Injury Research Centre, Faculty of Health and Behavioural Sciences, The University of Queensland, Brisbane 4072, Australia; d.theodoros@uq.edu.au (D.T.); l.cahill@uq.edu.au (L.C.); a.vaezipour@uq.edu.au (A.V.); 2 Faculty of Health and Behavioural Sciences, School of Health and Rehabilitation Sciences, The University of Queensland, Brisbane 4072, Australia; e.finch@uq.edu.au; 3 Centre for the Neuroscience of Speech, Department of Audiology and Speech Pathology, Melbourne School of Health Sciences, The University of Melbourne, Melbourne 3010, Australia; vogela@unimelb.edu.au; 4 Redenlab Inc., Melbourne 3000, Australia; 5 Centre for Functioning and Health Research, Metro South Hospital and Health Service, Queensland Health, Brisbane 4102, Australia; 6 The Princess Alexandra Hospital, Metro South Hospital and Health Service, Queensland Health, Brisbane 4102, Australia; anna.farrell@health.qld.gov.au; 7 The Royal Brisbane and Women’s Hospital, Metro North Hospital and Health Service, Queensland Health, Brisbane 4029, Australia; 8 Menzies Health Institute Queensland, School of Medicine and Dentistry, Griffith University, Gold Coast 4215, Australia; e.cardell@griffith.edu.au

**Keywords:** dysarthria, speech treatment, telerehabilitation, feasibility

## Abstract

This study evaluated the feasibility and outcomes of a telerehabilitation adaptation of the *Be Clear* speech treatment program for adults with non-progressive dysarthria to determine clinical delivery viability and future research directions. Treatment effects on speech clarity, intelligibility, communication effectiveness, and participation, as well as psychosocial outcomes in 15 participants with non-progressive dysarthria, were explored. Intervention involved daily 1-h online sessions (4 days per week for 4 weeks, totalling 16 sessions) and daily home practice. Outcome measures were obtained at baseline (PRE), post-treatment (POST), and 12 weeks following treatment (FUP). Feasibility measures targeting participant satisfaction, treatment adherence and fidelity, and technical viability were also employed. The programme was feasible concerning technical viability and implementation, treatment adherence and fidelity. High levels of participant satisfaction were reported. Increases in overall ratings of communication participation and effectiveness were identified at POST and FUP. Reductions in speech rate were identified at FUP. Improvements in aspects of lingual and laryngeal function were also noted after treatment. Over time, improvements relating to the negative impact of dysarthria were identified. Naïve listeners perceived negligible changes in speech clarity following treatment. Online delivery of the *Be Clear* speech treatment program was feasible, and some positive speech benefits were observed. Due to the small sample size included in this research, statistically significant findings related to speech outcomes must be interpreted with caution. An adequately powered randomised controlled trial of *Be Clear* online is warranted to evaluate treatment efficacy.

## 1. Introduction

Dysarthria is an acquired speech disorder causing reduced speech intelligibility due to weakened, imprecise, slow, and/or inco-ordinated movement of the muscles of speech motor control [1]. This motor speech disorder may present as a result of progressive (e.g., Parkinson’s Disease) or non-progressive (e.g., traumatic brain injury) neurological conditions [2]. It is a common and persistent sequela of acquired brain injury (ABI) in adulthood, with reported incidence rates as high as 60% [3]. A reduced ability to communicate as a consequence of dysarthria may result in adverse effects on self-esteem and personal relationships [4], and create barriers to recreation, employment, and educational opportunities [5]. 

Behavioural interventions delivered by speech-language pathologists (SLPs) represent the mainstay of treatment approaches for dysarthria management [6]. Interventions are influenced by the type and severity of the presenting speech deficit [1] and, specifically, the primary motor speech subsystem impairments contributing to reduced speech intelligibility. Given that dysarthria is a deficit in the neuromuscular execution of speech, intervention protocols typically involve training to increase physiological support for speech via exercises to increase the strength, range, precision, and speed of muscle movements involved in speech production [1,3]. Targets for these interventions may involve the articulators (e.g., lips and tongue), larynx, respiratory system, velopharynx, or any combination thereof, and may include speech and non-speech activities. Compensatory strategies may also be employed as an adjunct to the above approaches or as the preferred treatment. Compensatory strategies aim to maximise residual physiological function during communication exchanges [3]. 

While a general rehabilitative benefit of dysarthria treatment is accepted, the research evidence is constrained by a narrow scope of behavioural interventions evaluated with sufficient methodological rigour and equivocal findings in relation to speech outcomes in some studies [3]. A recent systematic review of speech treatment for non-progressive dysarthria in adults reported on 5 randomised control trials (RCTs) and 16 case series studies [6]. The largest body of evidence was derived from the use of the Lee Silverman Voice Treatment (LSVT-LOUD^®^), with 3 RCT studies [7,8,9] and 4 case series reports [10,11,12,13] indicating post-treatment improvements in speech intelligibility, articulatory precision, vowel space area, vocal loudness, and hypernasality, as well as the maintenance of some effects 4–6 months post-treatment. The findings of this review indicated that high-quality evidence for non-progressive dysarthria treatments that target motor speech subsystems other than laryngeal-respiratory systems is lacking and that further research is needed. 

The positive effects of LSVT LOUD^®^ on the speech of individuals with PD have been more widely documented than any other dysarthria treatment [14,15,16]. LSVT LOUD^®^ draws upon principles of motor learning (PML) that have been demonstrated to promote neural plasticity and brain reorganisation [17]. Principles of motor learning refer to conditions of practice and types of learning, such as practice structure (e.g., amount, distribution, variability, schedule, attentional focus, and target complexity) and nature of feedback (e.g., type, frequency, and timing) [18], that, when applied optimally, have the potential to enhance the brain’s recovery response. 

Recently, a novel treatment for adults with non-progressive dysarthria (*Be Clear*) incorporating the above principles of motor learning, neuroplasticity, and clear speech has been developed [19]. In this context, clear speech refers to the modification of habitual speech, resulting in enhanced intelligibility. The clear speech technique typically involves overarticulation and rate reduction as fundamental components [20]. *Be Clear* is an intensive speech treatment consisting of daily 1 h therapy sessions, 4 days per week, for a period of 4 weeks, and a self-directed daily home practice schedule of 15 min per day. The program utilises intensive and specific practice of salient speech production tasks aimed at improving the intelligibility of speech output. Following a small (*N* = 8) feasibility trial in adults with non-progressive dysarthria, the treatment schedule was reported as viable, acceptable, and appropriate for all participants, with significant improvements in speech intelligibility and overall communication function observed. Despite the small sample size and associated low statistical power of this study, positive outcomes reported within the context of a high-intensity protocol precipitated the exploration of an online adaptation of the program by the current authors.

Advances in information and communications technologies now permit in-home rehabilitation via telehealth. These services reduce barriers to accessing intensive treatment for those in rural and remote areas, as well as those with significant physical and/or communication impairments that find in-person treatment attendance challenging [21]. Furthermore, the global COVID-19 pandemic has highlighted that online service delivery is achievable and necessary to sustain rehabilitation programs when in-person contact is prohibited. 

Evidence supporting the use of telerehabilitation for managing dysarthria in adults is predominantly drawn from the treatment of hypokinetic dysarthria in PD via LSVT LOUD^®^ [22,23,24,25,26]. Notably, online treatment was found to be comparable to in-person treatment in relation to the delivery of LSVT LOUD^®^ via videoconferencing into participants’ homes [23]. This outcome was evidenced by significant improvements in acoustic, perceptual, and quality of life measures following treatment across randomised online and in-person treatment groups [23]. 

The current study aimed to evaluate the feasibility and outcomes of an online adaptation of the *Be Clear* speech treatment programme to determine clinical delivery viability and future research directions.

## 2. Materials and Methods

### 2.1. Design

The study utilised a rater-blinded, prospective, single cohort, repeated-measures (i.e., pre- treatment, immediately post-treatment, and 12 weeks following the completion of treatment) design. 

### 2.2. Participants

Participants were recruited from metropolitan speech pathology rehabilitation caseloads, as well as through social media promotion via brain injury support groups that encompassed both metropolitan and non-metropolitan areas (i.e., no more than a 2-h drive from the research site). A screening process was employed to ascertain eligibility for inclusion. Standard inclusion criteria for study participation were: (1) a diagnosis of non-progressive dysarthria as determined by an SLP experienced in dysarthria management; (2) be at least 6 months post-brain injury (i.e., outside the typical period of spontaneous motor recovery) [27]; (3) English as a native language; (4) not receiving/prepared not to receive additional speech pathology intervention for the duration of the study; (5) normal cognitive functioning as indicated by a score of ≥26/30 on the Montreal Cognitive Assessment (MoCA) [28]; and (6) normal performance (i.e., 15/15 items correct) on the Language Screening Test (LAST) to confirm intact expressive and receptive language abilities/absence of frank aphasia [29].

In addition to the above standard criteria, it was essential for participants to meet both speech stimulability and technical requirements. Successful stimulability testing was defined as the ability of a participant to produce clear speech following an SLP’s model. Clear speech techniques trialed with each participant included overarticulation, increased volume, and reduced speech rate. In relation to technical criteria, participants were required to have adequate internet connectivity within their homes to undertake online treatment. Connectivity status was determined during an in-person consultation with a clinician in the participant’s home prior to study inclusion via an internet speed test using Ookla (https://speedtest.net/ accessed on 4 April 2018). Depending on the telehealth platform employed for the delivery of treatment (i.e., eHAB or Coviu (see below for details)), the minimum upload and download speeds required ranged from 128 kilobits per second (kbps) to 350 kbps. Furthermore, participants were required to demonstrate basic computer skills (i.e., ability to respond to emails, use a mouse and keyboard) and, where possible, to have access to a personal desktop, laptop computer, phone, or tablet, with a webcam. For those participants without personal devices, a number of university iPads were available for loan for the duration of the treatment program. 

Fifteen participants (9 males and 6 females) with a diagnosis of non-progressive dysarthria after acquired brain injury (ABI) were recruited to the study, with ages ranging from 29 to 76 years (*M* = 52.67; *SD* = 12.97). Eight of the fifteen (8/15) participants had an underlying aetiology of traumatic brain injury, four participants developed dysarthria as a consequence of cerebrovascular accidents (CVA), 1 participant had a diagnosis of brain injury due to arteriovenous malformation, another participant due to encephalitis, and a further participant sustained a hypoxic brain injury during cardiac arrest. Dysarthria severity as determined by two SLPs experienced in dysarthria management (average years of experience = 15, SD = 0) ranged from mild to severe, in line with criteria on an informal 7 point rating scale (0= normal speech to 7 = severely unintelligible) [30]. Time post-injury at the commencement of treatment ranged from 10 to 44 months (M = 89.4, SD = 124.72). Overarticulation and increased volume were the clear speech techniques used by the participants in the current study. See Table 1 for participant characteristics, including dysarthria subtype as determined by the SLPs. Fifteen communication partners of the participants were also recruited to provide ratings of perceived communicative effectiveness post-treatment.

### 2.3. Procedure

Participants underwent communication evaluation that included the administration of perceptual speech assessments and the completion of everyday communication and quality of life questionnaires before and after online speech intervention. These evaluations were conducted on three (3) occasions at the following time intervals: at baseline (i.e., PRE); immediately post-treatment (i.e., POST = within 1 week subsequent to the completion of treatment); and at 12 weeks following the completion of treatment (i.e., FUP). Assessments were conducted in-person (i.e., in participants’ homes), consisting of two sessions across two days within the same week. Two SLPs (average years of experience = 15, SD = 0) were dedicated to assessment and data collection.

#### 2.3.1. Measures

Various measures were employed to evaluate the feasibility and outcomes of the online delivery of the *Be Clear* speech treatment program. Feasibility was examined by evaluating participant satisfaction, treatment adherence and fidelity, and the utility of technological adaptations. Speech/communication outcomes included: perceptual speech assessments, oromotor evaluation, assessments of everyday communication, and psychosocial impact of dysarthria. 

##### Feasibility Measures

Participant Satisfaction

A 13-question satisfaction survey using a 5-point Likert scale (i.e., 1 = strongly agree to 5 = strongly disagree) was administered to participants immediately post-treatment to ascertain perceptions of program quality, effectiveness, convenience, and ease of telerehabilitation.

Treatment adherence and fidelity

For each of the 16 treatment sessions, treating SLPs were provided with a session checklist of required treatment steps. This checklist was also used to record session absences and the completion of the previous day’s home practice tasks.

Technical feasibility

Treating SLPs were instructed to maintain a log of technical issues experienced on any occasion with all participants for the duration of the program. This information was written in a notes column included on each of the 16 session checklists provided for each participant. 

##### Outcome Measures

The below assessment battery incorporated 6 assessment sessions (2 at baseline, 2 immediately post-treatment, and 2 at follow-up) of approximately 2.5 h each. A total of approximately 7.5 h was required from each participant regarding data collection.

Perceptual assessments and oromotor function

Paired comparison ratings

Paired comparison ratings were employed to evaluate the effect of treatment on speech clarity, as judged by every day (i.e., naïve) listeners. Monologue speech samples of three minutes on a familiar topic were collected from participants at PRE, POST, and FUP. Speech samples were recorded using a condenser headset unidirectional microphone (AKG condenser model C420, AKG Acoustic, Vienna, Austria), positioned 5 cm from the participant’s mouth. Audio samples were captured by a Roland Duo-capture audio interface connected to a MacBook Air laptop using Audacity cross-platform audio software (version 2.2.2) [31]. All samples were recorded as .wav files, sampled at 44.1 kHz, and quantization at 16 bits. 

Samples of forty seconds were then prepared from each data point to form four paired monologue combinations per participant for analysis: 1) PRE-POST; 2) PRE-FUP; 3) POST-PRE; and 4) FUP-PRE. Ten adult naïve listeners (6 females and 4 males) listened to the paired speech samples using headphones. Raters were provided with 15 min of training using 10 randomly selected paired samples from the complete data set. Paired samples were then randomly presented to the listeners. Listeners were instructed to rate the second sample in each pair compared to the first sample, in terms of clarity/how easy the sample was to understand. The listeners were recruited from a pool of healthy adults involved in standardised patient programs across the university and were reimbursed for their efforts. Naïve listeners were native speakers of English, reported normal hearing, and had no prior exposure to dysarthric speech.

Assessment of Intelligibility of Dysarthric Speech

Speech intelligibility was evaluated using the Assessment of Intelligibility of Dysarthric Speech (ASSIDS) [32]. This assessment was administered twice at each evaluation time point (i.e., on 2 separate days) to accommodate day-to-day variability in speech production. Participants were required to read or repeat aloud a randomly selected list of 22 sentences chosen from the test manual. These speech samples were audio-recorded as per the recording procedure described previously. They were then prepared for analysis with numerical coding and randomly presented to two judges (blinded to the assessment condition) for transcription and timing of sentence duration. Intelligibility metrics, including sentence percentage intelligibility, words per minute (WPM), and communication efficiency ratio (CER), were calculated for each participant. 

Percentage sentence intelligibility was calculated by dividing the number of correctly transcribed words during the sentence reading task by the total number of words (i.e., 220). The speaking rate was determined by dividing the total number of words produced (i.e., 220) by speaking duration. The CER was calculated by dividing the rate of intelligible speech (i.e., number of correctly transcribed words/duration) by the average rate of normal speech (i.e., 190 words per minute). For statistical analysis, mean values (across judges and testing sessions) at each time point pertaining to the above metrics were used. 

Frenchay Dysarthria Assessment-2 (FDA-2)

The FDA-2 is a standardised evaluation of lip, tongue, velopharyngeal, laryngeal and respiratory functioning, coughing and swallowing reflexes, and speech intelligibility [33]. As speech intelligibility was independently assessed using the ASSIDS, these tasks were excluded from the FDA-2 administration in the current study. Participant performance was rated on the FDA-2′s 9-point alphabetical scale (i.e., a = normal function to e = no function). For statistical analysis, the alphabetic code was replaced with a numerical 9-point scale (e.g., a = 9; b.5 = 8; b = 7; c.5 = 6; c = 5; d.5 = 4; d = 3; e.5 = 2; e = 1). Group means across time points were used for statistical analysis in relation to six FDA-2 subtests and a composite score.

Everyday Communication

Communication Effectiveness Index- Modified (CETI-M)

Each participant and their communication partners were asked to individually complete a 10- item questionnaire (i.e., CETI-M) evaluating the effectiveness of the dysarthric participant’s communication across various contextual situations [34]. Each question was rated on a 7-point scale, ranging from 1 (not effective at all) to 7 (very effective). Participants were provided with a hard copy of the CETI-M upon completion of the first assessment session at each time point and were required to be completed prior to the second assessment session. Assistance from a caregiver where required was permitted.

Communication Participation Item Bank (CPIB)

The CPIB investigates the degree to which a participant’s speech impairment interferes with participation across a range of speaking environments (e.g., talking on the telephone, ordering in a restaurant) [35]. The short form of the CPIB was utilised in the current study and is comprised of 10 questions that are rated on a 4-point scale (i.e., 3 = Not at all, 2 = a little, 1 = quite a bit, 0 = Very much). Ratings were summed to produce a score ranging from 0 to 30. Higher scores indicate less interference to communication participation. Participants were also provided with a hard copy of the CPIB (as described above) upon completion of the first assessment session at each time point.

Dysarthria Impact Profile (DIP)

The DIP is a questionnaire designed to investigate the psychosocial impact of dysarthria from the speaker’s perspective [36]. It includes a total of 48 statements divided into five sections—Section A: The effect of dysarthria on me as a person; Section B: Accepting my dysarthria; Section C: How I feel others react to my speech; Section D: How dysarthria affects my communication with others; and Section E: Dysarthria relative to other worries and concerns. Participants are required to rate each statement on a 5-point scale from 1 = strongly agree to 5 = strongly disagree. These responses are given a weighted score (i.e., positively worded statements with which the respondent strongly agrees receive a score of 5, and statements strongly disagreed with receive a score of 1). In negatively worded statements, the reverse is true, with strongly disagree statements receiving a score of 5. These scores are then used to calculate a mean score per statement for each section, as well as a Total Impact Score (maximum of 225). Lower scores on this assessment are associated with a greater negative impact of dysarthria. Participants were provided with a hard copy of the DIP as described above.

#### 2.3.2. *Be Clear* Online Treatment

All participants commenced online speech treatment after baseline assessment. Details of the online *Be Clear* speech treatment program are described below, as per TIDieR guidelines [37]. Five SLPs experienced in dysarthria management (average years of experience = 19, SD = 4.18), provided individual online treatment to the study participants. One of these SLPs was also involved in data collection. None of the participants were assessed by their treating SLP. Each participant was allocated a dedicated treating SLP for the duration of the treatment program. The *Be Clear* online protocol aligned with the in-person protocol [19] regarding program components, tasks, and treatment duration. Specifically, online treatment was provided one hour per day, four days per week for 4 weeks (totalling 16 sessions). Each session comprised a pre-practice component, followed by a practice component incorporating functional phrases, service requests, and functional speech tasks. An essential element of the program involved participant ratings of speech clarity (on a scale of 1–10) concerning speech sample recordings captured and played back to participants during each session. Participants were also required to complete 15 min of home practice each day, including the rehearsal of functional phrases, service requests and functional speech tasks, and a transfer task (i.e., use of clear speech in a real-world communicative context). At the commencement of each session, the treating SLP checked in with each participant to confirm adherence with home practice tasks, and a written log pertaining to this information was maintained.

To determine the appropriate technology for an online adaptation of the *Be Clear* program, a task analysis was conducted. This analysis outlined treatment tasks, types of participant-clinician interactions, intervention stimuli, types of client responses, and necessary technical functionality for online delivery (see Table S1). This analysis revealed that a synchronous videoconferencing platform with the capacity to share intervention materials (text and images), as well as audio store and forward functionality, a whiteboard, and end-to-end encryption (private and secure communication), were required. 

Fit for purpose, readily available telehealth platforms were selected for the *Be Clear* online treatment program. Initially, the eHAB^®^ (Version 1.8.4) (a clinically validated telerehabilitation system) was selected given its functionalities for real-time videoconferencing, multi-device compatibility, and advanced media tools (including an audio store and forward application). Minimum upload and download requirement of 128 kbps was recommended for successful eHAB video conferencing. Six participants completed the program using eHAB. However, problems with audio playback of speech samples necessitated the selection of an alternative platform for the remaining 9 participants. Coviu (Coviu Global Pty Ltd., Sydney, Australia) was the alternative platform employed following the identification of issues with eHAB. Coviu is also a multi-device compatible, clinically validated telerehabilitation system, with live videoconferencing and audio store and forward functionality. For a 60-min one to one call via Coviu, a 900 MB bandwidth was required, with a minimum upload and download requirement of 350 kbps. All participants accessed telerehabilitation platforms via a WiFi-enabled device (i.e., iPad or computer). 

Pre-treatment training was conducted with the 5 treating SLPs. A group 2-h structured training session was conducted by research personnel (BMW) regarding the clear speech technique, program components, operation of the telerehabilitation systems, and creation and storage of resources. Documentation was provided to each treating clinician outlining the above, including individual session component checklists and a detailed outline of the original *Be Clear* treatment protocol [19]. Fidelity examiners (DT & BMW) conducted direct observations of treatment enactment with each treating clinician during one (1) treatment session within the first week of intervention, with a feedback provision. 

Online treatment was delivered to participants in their own homes or respite centre by trained SLPs. Before commencing treatment, all participants received a 1-h in-person structured training session in their homes, which included a live link to a treating clinician with opportunities to practice connection and experience online interaction and navigation of the telerehabilitation platform. For the treatment, participants connected via an email link or app, and were invited to join the call by the treating clinician who had full control of all telerehabilitation technology functions. Wherever possible, treatment sessions were scheduled for the same time each day and initiated via a videoconference call from the SLP at that time. Individually tailored treatment stimuli were pre-prepared in digital format for uploading during the sessions. Speech samples were randomly audio recorded throughout session tasks using store and forward applications and played back to participants to enable self-ratings of speech clarity (on a scale of 1 (unclear) to 10 (clear)). 

### 2.4. Data Analysis

Statistical analyses were conducted with IBM SPSS Statistics (Version 27 IBM Corp., Armonk, NY, USA). Descriptive statistics were used for the participant satisfaction, treatment adherence, fidelity, and technical feasibility data, as well as for the paired comparison ratings. Parametric and non-parametric statistical approaches accounted for interval and ordinal data sets. Fifteen participants were recruited at the commencement of the study, although one participant discontinued due to poor health at FUP, resulting in missing data for this time point. Multiple imputation methods were used to replace the missing data with the series mean for the missing data across follow-up outcome measures in SPSS. One-way repeated measures ANOVAs (with post-hoc pair-wise comparisons) were employed to determine time effects on percentage sentence intelligibility, words per minute, and communication efficiency ratios. Friedman’s two-way analyses of ranks were used to examine treatment effects on ordinal ratings of communication participation (i.e., CPIB) and effectiveness (i.e., CETI-M), Frenchay Dysarthria Assessment (i.e., FDA-2) performance, and the psychosocial impact of dysarthria (i.e., DIP). In the event of significant (*p* < 0.05) effects for time, post-hoc Wilcoxin Signed-rank tests using a stringent alpha level of *p* < 0.01 to account for the multiplicity of tests [38] were utilised to determine whether significant treatment effects occurred immediately POST and/or at FUP. Bonferroni procedures were not applied in this small cohort feasibility study, given the potential to further reduce power and increase Type II error rates to unacceptable levels [39]. 

Intraclass correlation coefficients (ICCs) and their 95% confidence intervals were calculated (absolute agreement, 2-way mixed-effects model) to determine inter- and intra-rater reliability for paired comparison and percentage sentence intelligibility ratings on the ASSIDS. The following criteria were used to determine the degree of inter- and intra-rater reliability: ICC values < 0.40 = poor to fair; 0.40 to 0.75 = moderate to good; > 0.75 = very good [40].

For the paired comparison ratings, inter-rater reliability was moderate across 10 listeners (ICC = 0.65, *p* < 0.001). Intra-rater reliability was determined by repeating 20% of the sample (*n* = 12) during the rating task, with six raters demonstrating very good reliability, two raters moderate reliability, and two raters exhibiting poor reliability (see Table S2). For the ASSIDs, very high inter-judge reliability was revealed across the 2 judges on the percentage sentence intelligibility subtest (ICC = 0.84, *p* = 0.001). Intra-judge reliability based on re-rating of 20% of the 88 sentence transcriptions (*n* = 18) revealed very high intra-judge reliability for both judges (i.e., Judge 1 (CC = 0.97, *p* < 0.001); Judge 2 (ICC = 0.87, *p* < 0.001).

## 3. Results

### 3.1. Feasibility

#### 3.1.1. Technical Feasibility

Treating SLP logs revealed that the first 6 study participants undertook the program via eHAB. Each of the treating clinicians (i.e., 3/3) reported technical issues with this platform. These issues included: connection difficulties, sub-optimal audio and video quality, and screen freezing. Issues were typically resolved via refreshing the website, restarting the session, or a computer restart. Issues with audio playback, however, were experienced. When successfully recorded, the quality of speech sample recordings for playback was poor, and on many occasions the audio recording function failed to record successfully. These issues necessitated a change of the telerehabilitation platform for the remaining participants.

Coviu enabled the successful delivery of the online speech treatment program, as reported by 3 out of 3 treating clinicians. Occasional connection issues were reported with the Coviu platform. Audio and video quality was largely reported as suitable, with occasional reductions in quality (e.g., freezing and pixilation). Few instances of display delay for onscreen resources were reported. The playback quality of audio recordings was reported to be poor on a few occasions, and, on two occasions, difficulty was reported with starting and stopping the audio recording function. In one session, the download and playback function was disabled. These issues were typically resolved via the refresh function, computer restart, or assistance from the Coviu help centre via online text chat. 

#### 3.1.2. Treatment Adherence and Fidelity

Thirteen of the fifteen (87%) participants completed the *Be Clear* online speech treatment program as per the original protocol [19] (i.e., 100% attendance with no alterations to the treatment schedule). Two participants completed 15/16 (94%) sessions, with non-attendance due to internet connectivity issues (1 participant) and power outage (1 participant), each on the final session. Make-up sessions were declined by each of these participants due to competing rehabilitation schedules. Speech outcome data for each of these participants were included in data analysis. A manual review of documentation checklists (BMW) at the completion of the study identified that 100% of treatment steps had been completed for attended sessions, across participants, except for participant judgements of speech clarity. Concerning the 6 participants who completed the program with eHAB, clarity ratings for speech sample recordings captured throughout sessions were not always possible due to technical malfunction. In this circumstance, feedback from the clinician was provided regarding the clarity of speech production, which included a rating between 1 and 10.

A sixteenth participant completed 7/16 treatment sessions (63%) in piecemeal fashion over a 3 week period and then withdrew from the study due to a lack of direct support worker assistance and an inability to adhere to program requirements. Subsequently, this participant’s data were excluded from analysis. Adherence to the home practice program was confirmed for all participants via self-report at the commencement of each online session and the FUP assessment.

#### 3.1.3. Participant Satisfaction

A post-treatment survey regarding participant experiences with the online treatment programme revealed a high level of satisfaction. Most participants indicated that online treatment was effective (see Figure S1), more convenient (see Figure S2) and that they felt comfortable communicating with the SLP via the internet (see Figure S3). All participants agreed that they could easily see and hear the SLP, that treatment via the internet saved travelling time, that they would be happy to receive online treatment again, and that they were happy with the quality of service provided via the internet. The majority of participants (14/15) agreed that receiving speech pathology services via the internet was acceptable (see Figure S4). Reports of the need for assistance to use the telerehabilitation system were balanced across the cohort, with 47% of participants stating that they required assistance, 47% stating that they did not require assistance, and 1 participant stating that they were unsure (see Figure S5). All but one participant (14/15) agreed that treatment via the internet would improve their quality of life, the remaining participant being unsure. Ten of the 15 participants (10/15) agreed that they would prefer to receive treatment via the internet rather than in-person (see Figure S6).

### 3.2. Therapeutic Effect

#### 3.2.1. Perceptual Assessments and Oromotor Function

##### Paired Comparison Ratings

Analysis of the 70 paired comparison ratings revealed that of the 36 samples comparing the pre- and immediately post-treatment monologue samples, 49% of the post-treatment samples and 43% of pre-treatment samples were identified as clearer or easier to understand (No Difference = 8%) by naïve listeners. Of the 34 ratings made comparing FUP with pre-treatment samples, 55% of the FUP samples and 40% of the pre-treatment samples were identified as clearer or easier to understand (No Difference = 5%).

##### Assessment of Intelligibility of Dysarthric Speech (ASSIDS)

A repeated-measures ANOVA indicated a main effect for time in relation to speech rate (WPM) (F = 3.83, *p* = 0.03) and communication efficiency ratio (CER) (F = 4.26, *p* = 0.02) (see Table 2). Post-hoc tests revealed statistically significant (*p* < 0.05) decreases in WPM at POST (*p* = 0.03) and FUP (*p* = 0.01), and in CER at FUP (*p* = 0.01). A main effect for time was not observed for percentage sentence intelligibility. 

##### Frenchay Dysarthria Assessment-2 (FDA-2)

A statistically significant increase (*X*^2^ = 13.93, *p* = 0.001) over time was determined for the composite scores achieved on the FDA-2. Post-hoc analysis revealed statistically significant increases in these scores between baseline and POST (*p* = 0.003) and between baseline and FUP (*p* = 0.003) (See Table 3). Significant main effects for time were also observed for some FDA-2 subsections: (1) Respiration (at rest) (*X*^2^ = 9.39, *p* = 0.01); (2) Laryngeal (pitch) (*X*^2^ = 8.34, *p* = 0.02); (3) Laryngeal (speech) (*X*^2^ = 8.34, *p* = 0.003); (4) Tongue (at rest) (*X*^2^ = 6.88, *p* = 0.03); (5) Tongue (protrusion) (*X*^2^ = 8.29, *p* = 0.02); (6) Tongue (speech) (*X*^2^ = 10.43, *p* = 0.008). Post-hoc analyses revealed statistically significant increases in scores on the Tongue (speech) subtest from baseline to POST (*p* = 0.008), and on the Tongue (protrusion) subtest from baseline to FUP (*p* = 0.008). Analyses failed to identify significant changes over time on the remaining FDA-2 subsections (see Table 3).

#### 3.2.2. Everyday Communication and Psychosocial Impact

##### Communication Effectiveness Index-Modified (CETI-M)

For the dysarthric speakers, the main effect regarding time was identified for 5 of the 10 CETI-M items, as well as for the total/summary score (see Table 4). More specifically, significant increases in total score (*X*^2^ = 6.15, *p* = 0.046) were observed, within the context of concurrent score increases over time on items 5 (*Having a conversation with a stranger over the phone*) (*X*^2^ = 7.64, *p* = 0.02), 7 (*Having a conversation with someone at a distance*) (*X*^2^ = 6.30, *p* = 0.04), 8 (*Having a conversation with someone in a noisy environment*) (*X*^2^ = 9.64, *p* = 0.01), 9 (*Speaking or having a conversation before a group*) (*X*^2^ = 7.45, *p* = 0.02), and 10 (*Having a long conversation with someone (over an hour)*) (*X*^2^ = 13.73, *p* = 0.001). Post hoc analyses revealed significant baseline to POST increases on Item 10 (*p* = 0.003). Baseline to FUP changes failed to reach significance (*p* < 0.01) on any item (see Table 4).

Caregivers’ (*n* = 15) perceptions of communication effectiveness revealed a main effect of time on all questions of the CETI-M and the Total Score (see Table 4). Post hoc analyses revealed significant (*p* < 0.01) baseline to POST increases on the Total score and Items 1, 2, 5–6, and 8. Significant (*p* < 0.01) baseline to FUP score increases were also observed on the Total Score, as well as on Items 2, 4, and 6–10 (see Table 4).

##### Communication Participation Item Bank (CPIB)

Significant changes over time on the CPIB were observed in relation to Items 4 (Does your condition interfere with communicating when you are out in your community (e.g., errands, appointments)?) (*X*^2^ = 11.49, *p* = 0.003), 5 (Does your condition interfere with asking questions in a conversation?) (*X*^2^ = 8.17, *p* = 0.02), 7 (Does your condition interfere with having a long conversation with someone you know about a book, movie, show, or sports event?) (*X*^2^ = 8.63, *p* = 0.01), 9 (Does your condition interfere with getting your turn in a fast-moving conversation?) (*X*^2^ = 9.14, *p* = 0.01) and the Summary Score (*X*^2^ = 11.47, *p* = 0.003). Post hoc analyses revealed significant (*p* < 0.01) baseline to POST increases on the Summary Score (*p* = 0.007) (see Table 5). Significant (*p* < 0.01) increases were also revealed from baseline to FUP on Item 4 and the Summary Score (see Table 5).

##### Dysarthria Impact Profile (DIP)

Significant (*p* < 0.05) changes across time were observed related to subsections A (Effect of dysarthria on me as a person), B (Accepting my dysarthria), C (How I feel others react to my speech), and D (How my dysarthria effects my communication with others) of the DIP (see Table 6). Post hoc analyses revealed significant baseline to POST increases in mean scores per statement in subsection D only (*p* = 0.003). At FUP, a significant increase from baseline in mean scores per statement was observed on subsection B only (see Table 6).

Given the small sample size investigated in this research, effect sizes were also calculated on total/composite scores obtained on the FDA-2, all qualitative measures and the three (3) ASSIDS metrics were employed (i.e., percent sentence intelligibility, words per minute, and communication efficiency ratio), to evaluate the magnitude of behavioural change and related clinical significance. Manual calculations to determine effect sizes related to the magnitude of change on the above metrics from both pre-treatment to post-treatment and pre-treatment to follow-up phases were undertaken using the following formula:

Mean_post or FUP_—Mean_pre_/SD_diff_ (1)

where SD_diff_ represented the standard deviation of paired differences. Large, moderate, or small effect sizes (*d*) were then identified as per standard criteria (i.e., large = 0.8; moderate = 0.5; small = 0.2) [41]. As represented in Table 7, strong effect sizes (i.e., ≥0.8) were observed across composite scores on all quality of life and functional communication measures (i.e., CETI-M, CPIB, DIP) and the FDA-2. This finding indicates a strong relationship between intervention and changes in oromotor function, communication effectiveness, participation, and psychosocial adjustment to dysarthria, supporting statistically significant findings. In contrast, small effects sizes (i.e., <0.5) were observed for each of the ASSIDS metrics at both POST and FUP time points (see Table 7). This finding suggests that behavioural changes identified on these metrics were of limited clinical significance. Although significant statistical reductions in CER and WPM on the ASSIDS were observed, these results must be interpreted with caution, given evidence of small effect sizes and the questionable clinical significance of post-treatment changes observed in relation to these variables. 

## 4. Discussion

In this study, the feasibility and outcomes of an online adaptation of the *Be Clear* speech treatment program were evaluated to determine clinical delivery viability and future research directions. Technical adaptations were readily available for online treatment implementation; however, technical difficulties were experienced with the audio store-and-forward function on one of the telerehabilitation platforms used with some participants. Overall, sessions were well attended, and participants reported a high level of satisfaction with online treatment and no statements of excessive burden in relation to assessment or intervention requirements. Participant recruitment was challenging, with 16 individuals recruited during a 20-month period. This may have been due to stringent exclusion criteria pertaining to concomitant aphasia and/or cognitive impairment. Future trials must consider this factor and determine whether these co-occurring diagnoses negatively impact the successful completion of online dysarthria intervention.

Positive outcomes were observed related to aspects of lingual and laryngeal function, perceptions of communication effectiveness and participation, and the psychosocial impact of dysarthria following treatment. Formal assessments of intelligibility revealed significant reductions in speech rate and communication efficiency ratios following treatment, although significant alterations in sentence intelligibility were not observed. Small effect sizes concerning post-treatment change on these ASSIDS metrics indicated that alterations in performance on these tasks may be of limited clinical significance, and therefore, cannot be confidently reported as treatment effects. In contrast, large effect sizes were identified in relation to post-treatment changes in total scores achieved on the FDA-2 and all quality of life and functional communication measures. This suggested that statistically significant changes on these measures may be considered meaningful treatment effects. Naïve listeners identified negligible differences in speech clarity when comparing pre- to post- treatment speech samples. These collective results indicated that the *Be Clear* speech treatment program could be viably implemented online for adults with non-progressive dysarthria. Preliminary findings suggest positive benefits associated with communication and quality of life outcomes following the *Be Clear* online intervention. However, a larger research trial is required to confirm these promising patterns in functional speech performance and the psychosocial impact of dysarthria.

### 4.1. Feasibility of Online Delivery

It is acknowledged that some technical challenges may have negatively impacted treatment adherence and fidelity in the current study. Connectivity issues, screen freezing, and display delays were successfully and expediently resolved via website refreshing, session restarting, computer restarting, and/or dedicated help centre assistance (where applicable). The initial six participants experienced issues with the audio store-and-forward function in eHAB required for participant self-ratings of speech clarity. An essential component of the in-person *Be Clear* treatment was the audio recording (via a digital audio recorder) and playback of intermittent speech samples for ratings of clarity during treatment sessions, in an effort to promote self-evaluation and thus generalisation of clear speech [19]. The inability to provide audio playback of speech samples with the six aforementioned participants was a fidelity risk in the current research that may have impacted treatment outcomes.

A work-around for this issue was additional prompting to participants on each task to focus on the clarity of their speech output, as well as the provision of verbal feedback from the clinician regarding the level of speech clarity achieved (in place of self-rating); however, this self-rating alternative was a deviation from the original protocol. Identifying a backup telehealth platform with matching functionality prior to treatment commencement to immediately accommodate this technical issue would have been advantageous. Audio store-and-forward functionality is not a standard within-meeting application in conventional videoconferencing platforms (e.g., Zoom and Skype). Without considering an identical, at-the-ready backup platform prior to treatment commencement, considerable time (i.e., approximately 2 weeks) was required to investigate alternative fit-for-purpose platforms while existing participants completed treatment via the substandard platform.

Treatment fidelity was also compromised by non-adherence with the original treatment protocol for two participants. Despite only completing 15 of 16 prescribed sessions, the data for these two participants was subsequently included in group statistical analysis, given the assumption that a therapeutic benefit would not have been compromised by the omission of a single (and final) session. It must be acknowledged, however, that this failure to strictly adhere to the original treatment plan may have negatively influenced outcomes for these participants [42].

Despite various technical issues across the research cohort, participants reported a high degree of satisfaction with the online delivery of *Be Clear.* Greater than 90% of study participants reported online treatment effectiveness, superior convenience to face-to-face treatment, comfort communicating online, and a willingness to receive online speech treatment again, indicating that online delivery was feasible. Two participants reported that they would have preferred to receive treatment in person. Further probing regarding the rationale for this survey response was not conducted in the current study. A preference for in-person treatment was not considered to reflect a study flaw, but perhaps preconception by some individuals that in-person treatment is more authentic and confidential, consistent with other behavioural research [43].

The attrition of a sixteenth participant in the current research due to a lack of support worker assistance to maintain the required treatment schedule indicated that physical support might be required by some individuals to successfully engage in intensive online speech treatment. The level of support required by some participants was most likely influenced by the presenting level of cognitive impairment and ability to independently adhere to a schedule, as well as prior experience with videoconferencing/videotelephony. Approximately half of the study cohort reported needing assistance to use the telerehabilitation system, and half reported that they did not require assistance. These findings indicate the need to consider and secure available caregiver support for participants during the implementation of online treatment for troubleshooting purposes.

### 4.2. Perceptual Assessments and Oromotor Function 

Participants in the current research demonstrated significant reductions in speech rate following treatment on the ASSIDS at both POST and FUP. This reduction in speech rate was anticipated by virtue of the clear speech techniques implemented in this treatment program. Kinematic and acoustic studies of articulatory displacement during loud and clear (i.e., overarticulated) speech have identified increases in articulatory movement patterns in normal speakers [44,45]. Although not a direct target of intervention in the current study, indirect reductions in rate during clear and loud speech may be attributed to increased articulatory displacement in these speaking conditions, necessitating increased time to produce more distinctive vocal tract shapes [46]. The results of the present study are consistent with other research that reports significant reductions in speech rate in dysarthric speakers subsequent to speech treatment programs incorporating clear and loud speech techniques [7,19,47]. Similarly, the significant reduction in CER observed at FUP can be explained by decreases in speech rate, given that this metric is calculated using intelligible words per minute produced relative to an average normative speech rate. Small effect sizes associated with WPM and CER changes following treatment, however, suggested that statistically significant findings in this small cohort may not be representative of treatment effects and as such should be interpreted cautiously.

In the area of dysarthria management, enhancing intelligibility is typically a primary goal for intervention [34]. Failure of the participants in the current study to demonstrate significant post-treatment increases in sentence intelligibility may be explained by several factors. On examination of baseline sentence intelligibility levels, the cohort achieved an average near ceiling (i.e., 90%) pre-treatment score that remained relatively stable at POST and FUP assessment. This profile suggests that although the study participants in this research were dysarthric, sentence intelligibility concerning a reading task (as measured via orthographic transcription) was relatively preserved before the commencement of treatment. It must be considered, therefore, that near ceiling reading-based intelligibility scores at baseline may offer redundant indices of treatment responsiveness in non-progressive dysarthria management [32]. When individual percentage sentence intelligibility profiles were examined in the current study, 10 out of 15 participants achieved baseline scores between 92 and 99.75%, with an average PRE-POST percentage change of 1.60% (range 0.25–2.5%). For the remaining 5 participants, baseline intelligibility scores ranged from 60–87%, with an average PRE-POST percentage change of 4.75% (range 0.75–10%). The participant with the lowest baseline score did not demonstrate the greatest degree of change, however, these profiles indicate that speakers with below ceiling baseline sentence intelligibility levels have a greater scope to increase the number of words correctly transcribed during reading tasks. A further point for consideration is that dysarthric speaker performance on a reading task does not consistently translate to intelligibility in conversational speech [48], nor does it provide an evaluation of speech functionality across contexts and partners [49]. Intelligibility ratings on such assessments may not be accurate predictors of functional speech intelligibility in everyday communicative contexts. 

Associated with near ceiling baseline scores and unremarkable post-treatment changes on transcription-based speech intelligibility measures was a predominance (i.e., 73%) of mild to moderate dysarthria severity levels in the current research cohort. Mild dysarthria and high scores on transcription-based intelligibility measures should not determine treatment candidacy. The experience of having dysarthria has been identified as equally significant across severity levels, indicating that even a mild deficit may negatively impact self-identity, social and emotional well-being, and perceptions of stigmatisation [4]. 

Paired comparison ratings indicated that naïve listeners were largely unable to detect differences in speech clarity/ease of understanding between PRE and POST and PRE and FUP speech samples in the current study. The listeners may have been influenced by participants’ reduced rate of speech post-treatment. The average speech rate for healthy speakers has been estimated at 190 words per minute [32]. Participants in the current research demonstrated a consistent decline in speaking rates across time that was approximately 50 percent slower than normal speech. Previous research indicates that speech spoken at slower than normal rates may be perceived as less natural than speech spoken at normal rates and that this perception is independent of intelligibility [50,51]. Naive listeners may have perceived slower post-treatment speech rates in the current study to be unnatural or irregular sounding, and therefore POST and FUP samples were not identified as clearer or easier to understand when compared to baseline samples. Similar to the percentage intelligibility findings above, high baseline intelligibility in the research cohort may have also precluded the perception of subtle changes in speech clarity following speech treatment, resulting in perceived speech changes at approximately chance level. A direct relationship between listener ratings of ease of understanding and speech intelligibility has been previously reported in dysarthria research [52]. In relation to treatment effects, changes in ease of understanding may not be easily detected when baseline intelligibility levels are high. 

The FDA-2 revealed significant post-treatment increases in composite test scores over time, largely attributed to improved performance on laryngeal and tongue subtests. Laryngeal and lingual subsystem improvements were not unanticipated, given the integration of loud and clear speech techniques within the treatment program. Treatment techniques such as increased loudness and overarticulation require increased respiratory-phonatory and articulatory effort, respectively [46,53]. This maximal effort is accompanied by increases in the timing, speed, and distance of articulatory movements [54]. Of note, clear and loud speech has been associated with multiple speech subsystem adjustments, including increased vocal intensity and quality, in concert with enhanced articulation [55,56,57,58,59]. These dual effects have been postulated to occur due to biomechanical and neurophysiological relationships between the articulatory and laryngeal subsystems [60]. Enhanced vocal quality and effectiveness and increased range and speed of lingual protrusion and articulation accuracy following online *Be Clear* treatment in the present research support the above findings.

### 4.3. Everyday Communication and Psychosocial Impact

Participants and their communication partners reported positive changes in everyday communication subsequent to speech treatment. In relation to overall post-treatment communication effectiveness, caregivers reported consistent improvement or maintenance of improved effectiveness over time on the CETI-M. Participants reported improved communication effectiveness immediately following treatment only concerning speaking stamina (i.e., engaging in lengthy conversations). In contrast, caregivers reported post-treatment improvements in communication effectiveness across 5 social contexts assessed by the CETI-M, as well as the Total score. At FUP, caregivers reported significant improvement in effectiveness across a greater number of social contexts, suggesting that positive effects of treatment in terms of functional gain endured and, in some domains, continued to improve well after the termination of treatment. Caregiver reports of long-term changes in post-treatment communicative effectiveness were consistent with other dysarthria treatment studies [61,62]. A similar profile was observed in relation to communication participation, with participant CPIB self-reports indicating an improved perceived ability to participate across a range of communicative contexts following treatment, which was typically maintained or demonstrated further improvement at FUP. 

Participants and caregivers identified improvements in functional intelligibility following speech treatment. These findings suggest positive treatment-related benefits in terms of communication effectiveness. The small sample size studied, however, and the inability of chosen speech intelligibility and clarity measures to detect post-treatment changes render these findings difficult to decipher. Communicative effectiveness has been identified as a discrete construct that may not be consistently related to dysarthria severity, or rather, a level of speech impairment [63]. Disconnects between speech intelligibility and functional communication performance are consistent with results reported in other dysarthria treatment studies [13,64]. Dysarthric speakers have previously identified discrepancies between speech quality and speech comprehensibility, with the perception that even though their speech sounds abnormal, they can be successfully understood by others with appropriate practice [4]. It may be argued, therefore, that communication effectiveness should represent the primary goal for treatment in the management of acquired dysarthria [65], and as such, related measures must be considered as crucial outcome measures in future dysarthria intervention research. 

In line with previous research, increased communicative effectiveness in the absence of changes to speech intelligibility and clarity in the present study may have resulted from the targeted use of clear speech strategies in real-life contexts and the relinquishment of these strategies within manufactured assessment environments [64]. In turn, successful communication exchanges within real-life contexts may have positively influenced communicative participation, with the resumption of previously abandoned social and recreational roles following online speech treatment [4]. Emerging research in dysarthria treatment highlights the importance of activity and participation treatment responsiveness in line with the World Health Organisation’s International Classification of Functioning, Disability and Health (ICF) framework [61]. Importantly, the recent introduction of such measures has indicated that speech treatment can improve the effectiveness of communication and social participation due to increased personal control over dysarthria and a reduction in the perceived communication handicap [61].

Concerning the psychosocial effects of online *Be Clear* treatment, participants demonstrated positive psychological and social adjustments to dysarthria following intervention across 4 sections of the DIP (i.e., Sections A–D). Immediately post-treatment, a significant improvement on Section D of the DIP (i.e., *How my dysarthria effects my communication with others*) was observed. Section D largely explores communication avoidance and participation restriction regarding the presenting dysarthria. The result indicated a direct improvement in the ability to actively communicate within a range of social contexts following treatment. At FUP, this significant increase in score on Section D was not maintained; however, a significant increase was observed related to scores achieved on Section B (i.e., *Accepting my dysarthria*) at FUP. Similar psychosocial adjustment profiles have been observed in other dysarthria treatment studies [65]. It may be argued that the above psychosocial adjustments may have resulted from the development of a therapeutic alliance, as alluded to in previous dysarthria treatment research [65]. Establishing a professional relationship with a clinician highly sensitive to the dysarthric speaker’s circumstances may have the potential to facilitate outcomes akin to direct intervention [1]. Caregiver corroboration of improved communication effectiveness in the current study, and indeed greater perceived improvement over a larger number of contexts following treatment, supports a probable treatment effect. Ongoing and/or maintained improvements in communication effectiveness three months after the termination of direct intervention suggests participant self-efficacy and the long-term implementation and success of clear speech strategies well after direct clinician-participant interactions had concluded. The observation of large effect sizes for post-treatment changes on total scores obtained on the FDA-2 and all quality of life and functional communication measures suggest that the statistically significant improvements observed regarding these measures may represent treatment effects.

## 5. Limitations and Directions for Future Research

Despite observing positive changes in oromotor function, everyday communication, and psychosocial adjustment to dysarthria following *Be Clear* online treatment, several study limitations were identified. Firstly, technical issues experienced during treatment implementation questioned the strength of the speech outcomes reported in this research. The evident failure of the audio store-and-forward function within the original telerehabilitation platform employed in this research presented a risk to treatment fidelity. Ensuring backup equipment with matching functionality prior to future clinical outcomes studies is recommended to mitigate this risk. Finally, non-adherence to the planned treatment dosage (i.e., missed sessions) did not exclude participant data from analysis in this study. It is acknowledged, however, that reduced dosage may have negatively impacted behavioural performance. 

Concerning selected outcome measures, the incorporation of acoustic analyses may have identified subtle changes in the speech signal following treatment that were undetectable via traditional intelligibility measures and naïve listener speech sample ratings in the current research. This finding highlights the inadequacy of dysarthria outcome measures in some contexts, and the need to develop more objective speech recognition tools for use in everyday communication contexts that may have the capacity to detect changes in the speech signal during functional communication activities.

The findings reported in this study must be interpreted within the context of a single cohort non-randomised feasibility study. Large-scale randomised controlled trials (RCT) represent a focus for future research to validate the efficacy of *Be Clear* online. Incorporation of face-to-face and online *Be Clear* groups within future RCTs, as well as usual care and waitlist control groups are anticipated, notwithstanding known recruitment challenges in vulnerable groups. A multicentre approach incorporating hospital, community health, and private speech pathology sectors is foreseen, with trial-specific training of recruitment staff essential. Outcome measures in future research will focus heavily on functional intelligibility. Traditional transcription-based intelligibility measures should also be evaluated in a larger trial. Alternative perceptual ratings of dysarthria severity, the naturalness of speech, and other speech signal parameters from speech samples collected during assessment sessions and functional communication exchanges should also be considered in an effort to identify what aspects of the speech signal directly influence communication effectiveness in functional contexts. 

## 6. Conclusions

The results of the current research provide preliminary evidence to support the online implementation of the *Be Clear* speech treatment program as a viable service delivery option for adults with non-progressive dysarthria. The most notable post-treatment improvements observed related to everyday communication and psychosocial dysarthria adjustment measures that were maintained or continued to increase three months following treatment completion. Notwithstanding individual cohort differences in terms of sample size, dysarthria severity, baseline intelligibility, and outcome measures employed, the findings of the current research were consistent with the original face-to-face *Be Clear* study [19], and indicate that a large-scale RCT is needed to establish treatment efficacy.

## Figures and Tables

**Table 1:** Participant characteristics.

Case	Sex	Age	Etiology	Dysarthria Type	Dysarthria Severity	Time Post Onset (Months) ^a^	Clear Speech Strategy
1	M	59	CVA	Flaccid-ataxic	Mild	13	overarticulation
2	F	44	TBI/MVA	Spastic-flaccid	Severe	26	overarticulation
3	M	43	TBI/MVA	Spastic-hypokinetic	Mild-moderate	17	overarticulation
4	M	45	TBI	Ataxic-flaccid	Moderate- severe	12	overarticulation
5	F	76	CVA	Spastic-hypokinetic	Mild- moderate	72	increased volume
6	M	71	AVM	Flaccid-ataxic	Mild	10	overarticulation
7	F	52	CVA	Spastic-ataxic	Mild-moderate	120	overarticulation
8	F	53	Encephalitis	Ataxic	Mild	27	overarticulation
9	F	42	TBI/Fall	Spastic-ataxic	Mild	31	overarticulation
10	M	66	CVA	(L) UUMN	Mild-moderate	13	overarticulation
11	M	37	TBI/Fall	Spastic	Moderate	90	overarticulation
12	M	29	TBI/MVA	Spastic-flaccid	Moderate-severe	96	overarticulation
13	F	54	TBI/MVA	Spastic-flaccid	Moderate-severe	444	increased volume
14	M	62	Hypoxic brain injury	Spastic	Mild	34	overarticulation
15	M	57	TBI	Spastic	Moderate	312	overarticulation

Note. AVM = arteriovenous malformation; CVA = cerebrovascular accident; F = female; L= left; M = male; MVA = motor vehicle accident; TBI = traumatic brain injury; UUMN = unilateral upper motor neuron. ^a^ Indicates time post-onset at the commencement of speech treatment.

**Table 2:** Assessment of Intelligibility of Dysarthric Speech (ASSIDS).

Subtest	Pre-Treatment M (SD)	Post-Treatment M (SD)	Follow-Up M (SD)	Time Main Effect		Pair-Wise Comparisons	
				*F*	*p*	Pre-Post *p*	Pre-FUP *p*
% Sentence Intelligibility	89.75 (13.20)	91.03 (12.75)	89.16 (14.89)	2.88	0.06	N/A	N/A
WPM	95.86 (17.47)	92.42 (16.99)	91.92 (15.49)	3.83	0.03 *	0.03 *	0.01 *
CER	0.46 (0.12)	0.45 (0.11)	0.43 (0.11)	4.26	0.02 *	0.19	0.01 *

Note: WPM = words per minute; CER = Communication Efficiency Ratio; N/A = not applicable; * Statistically significant at *p* < 0.05.

**Table 3:** Frenchay dysarthria assessment-2 (FDA-2) Results.

Function	Pre M (SD)	Post M (SD)	FUP M (SD)	Time Main Effect		Post Hoc Contrasts	
				*X* ^2^	*p*	Pre-Post *p*	Pre-FUP *p*
Cough	6.80 (1.86)	6.87 (1.73)	6.86 (1.64)	0.08	0.96	-	-
Swallow	6.67 (1.84)	6.80 (1.74)	6.93 (1.62)	1.24	0.54	-	-
Dribble/Drool	6.73 (2.19)	7.07 (1.67)	7.00 (1.65)	1.37	0.50	-	-
Respiration—at rest	5.93 (2.12)	6.87 (2.00)	6.93 (1.62)	9.39	0.01 *	0.03	0.03
Respiration—in speech	6.40 (1.88)	6.73 (2.05)	6.86 (1.92)	3.15	0.21	-	-
Lips—at rest	6.80 (2.08)	7.13 (2.17)	7.00 (1.96)	1.36	0.51	-	-
Lip—spread	6.73 (1.28)	7.00 (1.60)	6.93 (1.39)	0.29	0.87	-	-
Lip—seal	7.07 (1.53)	7.47 (1.51)	7.64 (1.23)	3.59	0.17	-	-
Lips—alternating motion	6.60 (1.45)	6.73 (1.39)	6.79 (1.37)	0.54	0.76	-	-
Lip—in speech	7.33 (1.05)	7.60 (1.12)	7.57 (1.05)	3.20	0.20	-	-
Palate—fluids	8.13 (1.25)	8.20 (1.32)	8.43 (1.12)	1.08	0.58	-	-
Palate—maintenance	7.80 (2.08)	7.93 (2.05)	7.86 (2.03)	0.70	0.71	-	-
Palate—in speech	7.80 (1.82)	8.07 (1.79)	7.93 (1.79)	2.80	0.25	-	-
Laryngeal—time	5.80 (2.65)	6.40 (2.64)	6.36 (2.41)	1.36	0.51	-	-
Laryngeal—pitch	4.87 (2.26)	5.67 (2.26)	5.86 (2.20)	8.34	0.02 *	0.05	0.04
Laryngeal—volume	4.80 (2.24)	5.20 (2.08)	5.43 (1.68)	4.61	0.10	-	-
Laryngeal—in speech	5.47 (1.69)	6.20 (2.01)	6.14 (1.88)	11.84	0.003 *	0.01	0.02
Tongue—at rest	7.00 (2.07)	7.20 (1.94)	7.64 (1.49)	6.88	0.03 *	0.56	0.03
Tongue—protrusion	6.27 (1.28)	6.47 (1.46)	7.29 (1.22)	8.29	0.02 *	0.54	0.008 **
Tongue—elevation	5.53 (1.85)	5.67 (2.13)	6.29 (2.08)	1.65	0.44	-	-
Tongue—lateralisation	5.93 (1.22)	6.60 (1.35)	7.00 (1.41)	4.44	0.11	-	-
Tongue—alternating motion	6.13 (0.92)	6.27 (1.16)	6.43 (1.24)	1.93	0.38	-	-
Tongue—in speech	6.47 (1.25)	7.13 (1.13)	7.07 (1.10)	10.43	0.01 *	0.008 **	0.02
Composite score	149.07 (23.34)	157.27 (27.40)	160.21 (26.37)	13.93	0.001 *	0.003 **	0.003 **

* Statistically significant at *p* < 0.05, ** Statistically significant at *p* < 0.01.

**Table 4:** Communication Effectiveness Index Modified (CETI-M).

Question	Dysarthric Speakers (*n* = 15)			Time Main Effect		Post-Hoc Contrasts		Caregivers (*n* = 15)			Time Main Effect		Post-Hoc Contrasts	
	PRE M (SD)	POST M (SD)	FUP M (SD)	*Χ* ^2^	*p*	PRE-POST *p*	PRE-FUP *p*	PRE M (SD)	POST M (SD)	FUP M (SD)	*Χ* ^2^	*p*	PRE-POST *p*	PRE-FUP *p*
Having a conversation with…														
1. a familiar person in a quiet environment	5.47 (1.13)	5.80 (1.08)	5.71 (1.28)	1.65	0.44	-	-	5.13 (1.46)	5.87 (1.13)	5.79 (1.15)	9.18	0.01 *	0.008 **	0.03
2. strangers in a quiet environment	4.33 (1.76)	5.20 (1.52)	5.14 (1.46)	4.04	0.13	-	-	3.93 (1.83)	5.07 (1.34)	5.14 (1.19)	11.78	0.003 *	0.006 **	0.005 **
3. a familiar person over the phone	4.40 (1.88)	5.27 (1.71)	5.50 (1.40)	3.80	0.15	-	-	4.47 (1.10)	5.47 (1.60)	5.50 (1.30)	8.97	0.01 *	0.014	0.012
4. young children	3.73 (1.71)	5.00 (1.60)	4.79 (1.74)	4.33	0.12	-	-	3.80 (1.78)	4.67 (1.35)	4.86 (1.36)	8.85	0.01 *	0.02	0.009 **
5. a stranger over the phone	3.47 (2.03)	4.33 (1.88)	4.29 (1.87)	7.64	0.02 *	0.05	0.16	3.33 (2.19)	4.80 (1.37)	4.57 (1.50)	10.08	0.01 *	0.009 **	0.02
6. while travelling in the car	4.20 (1.57)	4.73 (1.87)	4.29 (1.71)	1.44	0.49	-	-	3.73 (1.71)	4.87 (1.19)	4.93 (1.53)	15.49	0.001 *	0.004 **	0.003 **
7. someone at a distance	3.00 (1.51)	4.27 (1.71)	4.00 (1.46)	6.30	0.04 *	0.01	0.04	2.80 (1.70)	3.87 (1.55)	4.00 (1.73)	10.84	0.004 *	0.02	0.007 **
8. someone in a noisy environment	2.60 (1.81)	3.73 (1.49)	3.64 (1.63)	9.64	0.01 *	0.01	0.01	2.73 (1.58)	4.07 (1.49)	4.14 (1.51)	13.59	0.001 *	0.003 **	0.003 **
9. before a group	3.33 (1.76)	4.07 (1.79)	4.36 (1.63)	7.45	0.02 *	0.07	0.03	3.13 (1.51)	4.20 (1.47)	4.36 (1.63)	9.96	0.01 *	0.02	0.008 **
10. someone (over an hour)	3.13 (1.77)	4.47 (1.73)	4.57 (1.68)	13.73	0.001 *	0.003 **	0.01	2.87 (1.77)	3.93 (1.58)	4.50 (1.55)	13.50	0.001 *	0.03	0.001 **
Total Score	37.67 (13.11)	46.87 (12.40)	46.29 (11.73)	6.15	0.046 *	0.01	0.01	35.93 (15.32)	46.80 (11.61)	47.79 (12.50)	16.24	0.001 *	0.002 **	0.001 **

Note: PRE = pre-treatment; POST = post treatment; FUP = follow-up treatment.* Statistically significant at *p* < 0.05, ** Statistically significant at *p* < 0.01.

**Table 5:** Communication Participation Item Bank (CPIB).

Question Does Your Condition Interfere with...	Pre-Treatment M (SD)	Post-Treatment M (SD)	Follow-Up M (SD)	Time Main Effect		Post-Hoc Contrasts	
				*Χ* ^2^	*p*	Pre-Post *p*	Pre-FUP *p*
1. talking with people you know?	2.87 (0.83)	3.00 (0.85)	3.36 (0.72)	4.67	0.10	-	-
2. communicating when you need to say something quickly?	2.13 (0.92)	2.33 (0.98)	2.29 (1.03)	0.05	0.98	-	-
3. talking with people you do not know?	2.20 (0.86)	2.67 (1.18)	2.57 (1.05)	4.92	0.09	-	-
4. communicating when you are out in your community?	2.33 (0.82)	3.00 (0.85)	3.07 (0.80)	11.49	0.003 *	0.02	0.004 **
5. asking questions in a conversation?	2.47 (0.83)	3.20 (0.78)	3.00 (0.76)	8.17	0.02 *	0.01	0.06
6. communicating in a small group of people?	2.47 (0.92)	2.87 (0.74)	2.63 (0.70)	4.00	0.14	-	-
7. having a long conversation with someone you know?	2.47 (0.92)	3.00 (0.76)	3.07 (0.70)	8.63	0.01 *	0.02	0.02
8. giving someone detailed information?	2.07 (0.80)	2.53 (0.92)	2.57 (0.90)	5.35	0.07	-	-
9. getting your turn in a fast moving conversation?	1.67 (0.82)	2.40 (1.12)	2.36 (0.90)	9.14	0.01 *	0.02	0.01
10. trying to persuade a friend or family member to see a different point of view?	2.33 (0.98)	2.87 (1.19)	2.79 (0.86)	3.17	0.21	-	-
Summary Score	21.00 (6.60)	25.93 (7.52)	28.00 (6.77)	11.47	0.003 *	0.007 **	0.03 **

* Statistically significant at *p* < 0.05, ** Statistically significant at *p* < 0.01.

**Table 6:** Dysarthria Impact Profile (DIP) Results.

DIP Section	Pre-Treatment M (SD)	Post-Treatment M (SD)	Follow-Up M (SD)	Time Main Effect		Post-Hoc Contrasts	
				*Χ* ^2^	*p*	Pre-Post *p*	Pre-FUP *p*
A. Effect of dysarthria on me as a person	2.67 (1.29)	2.79 (1.35)	2.94 (1.26)	8.71	0.01 *	0.14	0.05
B. Accepting my dysarthria	3.11 (1.36)	3.23 (1.33)	3.51 (1.27)	8.26	0.02 *	0.52	0.001 **
C. How I feel others react to my speech	3.08 (1.30)	3.19 (1.28)	3.29 (1.29)	8.96	0.01 *	0.23	0.01
D. How dysarthria affects my communication with others	3.19 (1.33)	3.51 (1.24)	3.44 (1.27)	14.35	0.001 *	0.003 **	0.01
E. Dysarthria relative to other worries and concerns	2.73 (1.53)	2.60 (1.50)	2.79 (1.42)	0.21	0.90	-	-
Total Impact Score	146.60 (23.10)	154.63 (20.70)	160.79 (29.91)	5.20	0.07	-	-

* Statistically significant at *p* < 0.05, ** Statistically significant at *p* < 0.01.

**Table 7:** Effect sizes for total scores across outcome measures.

	Pre-Treatment *M*	Post -Treatment *M*	FUP *M*	SD_diff1_	SD_diff2_	*d* ^1^	*d* ^2^
CETI-M dys^TOT^	37.67	46.87	46.27	11.75	10.6	0.8	0.8
CETI-M CG ^TOT^	35.93	46.8	47.8	9.59	8.79	1.1	1.4
CPIB ^TOT^	21.0	25.93	28.0	5.89	6.59	0.9	1.1
DIP ^TOT^	146.6	154.6	160.8	13.94	16.42	0.6	0.9
FDA-2 ^TOT^	149.07	157.27	160.2	8.34	11.68	1.0	1.0
ASSIDS % ^TOT^	89.75	91.03	89.16	5.89	6.40	0.2	0.09
ASSIDS WPM ^TOT^	95.86	92.42	91.92	11.74	11.36	0.3	0.3
ASSIDS CER ^TOT^	0.46	0.45	0.43	0.06	0.06	0.2	0.4

Note. CETI-M dys TOT = CETI-M dysarthria speaker total; CETI-M caregiver TOT = CETI-M caregiver total; CPIB TOT = Communication Participation Item Bank total; DIP TOT = Dysarthria Impact Profile Total; FDA-2 TOT = Frenchay Dysarthria Assessment-2 total; ASSIDS % TOT = % sentence intelligibility total; ASSIDS WPM TOT = words per minute total; ASSIDS CER TOT = communication efficiency ratio total; SD_diff1_ = standard deviation of paired PRE-POST differences; SD_diff2_ = standard deviation of paired PRE-FUP differences; *d*^1^ = effect size in relation to magnitude of PRE-POST change; *d*^2^ = effect size in relation to magnitude of PRE-FUP change.

## Data Availability

The data presented in this study is available on request.

## Supplementary Materials

The following are available online at https://www.mdpi.com/article/10.3390/brainsci12020197/s1, Table S1: *Be Clear* online task analysis; Table S2: Intra-rater reliability intraclass correlations (ICC) for 10 naïve listeners; Figure S1: Participant ratings of online treatment effectiveness; Figure S2: Participant ratings of online treatment convenience; Figure S3: Participant ratings of comfortableness with online treatment; Figure S4: Participant ratings of online treatment acceptability; Figure S5: Participant ratings of required telerehabilitation assistance; Figure S6: Participant preferences for service delivery.

## References

[1] Duffy J. (2013). Motor Speech Disorders: Substrates, Differential Diagnosis, and Management.

[2] Magee M., Copland D., Vogel A.P. (2019). Motor speech and non-motor endophenotypes of Parkinson’s disease. Expert Rev. Neurother..

[3] Mitchell C., Bowen A., Tyson S., Butterfint Z., Conroy P. (2017). Interventions for dysarthria due to stroke and other adult-acquired, non-progressive brain injury. Cochrane Database Syst. Rev..

[4] Dickson S., Barbour R.S., Brady M., Clark A., Paton G. (2008). Patients’ experiences of disruptions associated with post-stroke dysarthria. Int. J. Lang. Commun. Disord..

[5] Enderby P. (2013). Disorders of Communication: Dysarthria. Handb. Clin. Neurol..

[6] Finch E., Rumbach A.F., Park S. (2020). Speech pathology management of non-progressive dysarthria: A systematic review of the literature. Disabil. Rehabil..

[7] Wenke R., Theodoros D.G., Cornwell P. (2011). A comparison of the effects of the Lee Silverman voice treatment and traditional therapy on intelligibility, perceptual speech features, and everyday communication in nonprogressive dysarthria. J. Med. Speech Lang. Pathol..

[8] Wenke R., Theodoros D.G., Cornwell P. (2010). Effectiveness of Lee Silverman Voice Treatment (LSVT)® on hypernasality in non-progressive dysarthria: The need for further research. Int. J. Lang. Commun. Disord..

[9] Wenke R., Theodoros D.G., Cornwell P. (2010). Changes to articulation following LSVT® and traditional dysarthria therapy in non-progressive dysarthria. Int. J. Speech Lang. Pathol..

[10] Mahler L.A., Jones H.N. (2012). Intensive treatment of dysarthria in two adults with Down syndrome. Dev. Neurorehabilit..

[11] Mahler L.A., Ramig L.O. (2012). Intensive treatment of dysarthria secondary to stroke. Clin. Linguist. Phon..

[12] Mahler L.A., Ramig L.O., Fox C. (2009). Intensive voice treatment (LSVT [R] LOUD) for dysarthria secondary to stroke. J. Med. Speech Lang. Pathol..

[13] Wenke R., Theodoros D.G., Cornwell P. (2008). The short- and long-term effectiveness of the LSVT for dysarthria following TBI and stroke. Brain Inj..

[14] Ramig L.O., Halpern A., Spielman J., Fox C., Freeman K. (2018). Speech treatment in Parkinson’s Disease: Randomized control trial. Mov. Disord..

[15] Levy E., Moya-Gale G., Chang Y., Freeman K., Forrest K., Brin M.F., Ramig L.O. (2020). The effects of intensive speech treatment on intelligibility in Parkinson’s disease: A randomised controlled trial. EClinicalMedicine.

[16] Atkinson-Clement C., Sadat J., Pinto S. (2015). Behavioural treatments for speech in Parkinson’s disease: Meta-analyses and review of the literature. Neurodegener. Dis. Manag..

[17] Ludlow C.L., Hoit J., Kent R., Ramig L.O., Shrivastav R., Strand E., Sapienza C.M. (2008). Translating principles of neural plasticity into research on speech motor control recovery and rehabilitation. J. Speech Lang. Heart Res..

[18] Mass E., Robin D.A., Austerman Hula S.N., Wulf G., Ballard K., Schmidt R.A. (2008). Principles of motor learning in treatment of motor speech disorders. Am. J. Speech-Lang. Pathol..

[19] Park S., Theodoros D., Finch E., Cardell E. (2016). *Be Clear*: A new intensive speech treatment for adults with non-progressive dysarthria. Am. J. Speech-Lang. Pathol..

[20] Picheny M.A., Durlach N.I., Braida L.D. (1986). Speaking clearly for the hard of hearing. II: Acoustic characteristics of clear and conversational speech. J. Speech Heart Res..

[21] Theodoros D. (2012). A new era in speech-language pathology practice: Innovation and diversification. Int. J. Speech Lang. Pathol..

[22] Constantinescu G., Theodoros D.G., Russell T., Ward E.C., Wilson S., Wootton R. (2011). Treating disordered speech and voice in Parkinson’s disease online: A randomised controlled non-inferiority trial. Int. J. Lang. Commun. Disord..

[23] Theodoros D.G., Hill A.J., Russell T.G. (2016). Clinical and quality of life outcomes of speech treatment for Parkinson’s disease delivered to the home via telerehabilitation: A noninferiority randomised controlled trial. Am. J. Speech-Lang. Pathol..

[24] Constantinescu G., Theodoros D.G., Russell T.G., Ward E., Wilson S., Wooton R. (2010). Home-based speech treatment for Parkinson’s disease delivered remotely: A case report. J. Telemed. Telecare.

[25] Tindall L.R., Heubner R.A. (2009). The impact of an application of telerehabilitation technology on caregiver burden. Int. J. Telerehabil..

[26] Tindall L.R., Heubner R.A., Stemple J.C., Kleinert H.L. (2008). Videophone-delivered voice therapy: A comparative analysis of outcomes to traditional delivery for adults with Parkinson’s disease. Telemed. Ehealth.

[27] Cassidy J.M., Cramer S.C. (2017). Spontaneous and therapeutic-induced mechanisms of functional recovery after stroke. Transl. Stroke Res..

[28] Nasreddine Z.S., Phillips N.A., Bedirian V., Charbonneau S., Whitehead V., Collin I., Cummings J.L., Chertkow H. (2005). The Montreal Cognitive Assessment (MoCA): A brief screening tool for mild cognitive impairment. J. Am. Geriatr. Soc..

[29] Flamand-Roze C., Falissard B., Roze E., Maintigneux L., Beziz J., Chacon A., Join-Lambert C., Adams D.H., Denier C. (2011). Validation of a new language screening tool for patients with acute stroke: The Language Screening Test (LAST). Stroke.

[30] Darley F.L., Aronson A.E., Brown J.R. (1969). Differential diagnostic patterns of dysarthria. J. Speech Heart Res..

[31] Team A.D. (2018). Audacity.

[32] Yorkston K.M., Beukelman D.R., Traynor C. (1984). Assessment of Intelligibility of Dysarthric Spech.

[33] Enderby P., Palmer R. (2008). FDA-2: Frenchay Dysarthria Assessment.

[34] Yorkston K.M., Beukelman D.R., Strand E., Bell K.R. (1999). Management of Motor Speech Disorders in Children and Adults.

[35] Baylor C., Yorkston K., Eadie T., Kim J., Chung H., Amtmann D. (2012). The Communicative Participation Item Bank (CPIB): Item Bank Calibration and Development of a Disorder-Generic Form. J. Speech Lang. Heart Res..

[36] Walshe M., Peach R., Miller N. (2009). Dysarthria impact profile: Development of a scale to measure psychosocial effects. Intern. J. Lang. Commun. Disord..

[37] Hoffman T.C., Glasziou P.P., Boutron I., MIlne R., Perera R., Moher D., Altman D.G., Barbour V., Macdonald H., Johnston M. (2014). Better reporting of interventions: Template for intervention description and replication (TIDieR) checklist and guide. BMJ.

[38] Armstrong R.A. (2014). When to use the Bonferroni correction. Ophthalmic Physiol. Opt..

[39] Nakagawa S. (2004). A farewell to Bonferroni: The problems of low statitsical power and publication bias. Behav. Ecol..

[40] Fleiss J.L. (1981). Statistical Methods for Rates and Proportions.

[41] Cohen J. (1988). Statistical Power Analysis for the Behavioural Sciences.

[42] Jin J., Skiar G.E., Oh V.M.S., Li S.C. (2008). Factors affecting therapeutic compliance: A review from the patient’s perspective. Ther. Clin. Risk Manag..

[43] Renn B.N., Hoeft T.J., Lee H.S., Bauer A.M., Arean P.A. (2019). Prefernce for in-person psychotherapy versus digital psychotherapy options for depression: Survey of adults in the US. NPJ Digit. Med..

[44] Schulman R. (1989). Articulatory dynamics of loud and normal speech. J. Acoust. Soc. Am..

[45] Tasko S., McClean M. (2004). Variations in articulatory movement with changes in speech task. J. Speech Lang. Heart Res..

[46] Tjaden K., Sussman J.E., Wilding G.E. (2014). Impact of clear, loud, and slow speech on scaled intelligibility and spech severity in Parkinson’s disease and multiple sclerosis. J. Speech Lang. Heart Res..

[47] Sapir S., Spielman J., Ramig L.O., Hinds S.L., Countryman S., Fox C., Story B. (2003). Effects of intensive voice treatment (the Lee Silverman Voice Treatment (LSVT)) on ataxic dysarthria: A case study. Am. J. Speech-Lang. Pathol..

[48] Tjaden K., Wilding G. (2011). Effects of speaking task on intelligibility in Parkinson’s disease. Clin. Linguist. Phon..

[49] Hustad K.C. (2006). Estimating the intelligibility of speakers with dysarthria. Folia Phoniatr Logop..

[50] Dagenias P., Brown G., Moore R. (2006). Speech rate effects upon intelligibility and acceptability of dysarthric speech. Clin. Linguist. Phon..

[51] Hanson E.K., Beukelman D.R., Fager S., Ullman C. (2004). Listener attitudes toward speech supplementation strategies used by speakers with dysarthria. J. Med. Speech Lang. Pathol..

[52] Landa S., Pennington L., Miller N., Robson S., Thompson V., Steen N. (2014). Association between objective measurement of the speech intelligibility of young people with dysarthria and listener ratings of ease of understanding. Int. J. Speech Lang. Pathol..

[53] Lindblom B. (1990). Explaining Phonetic Variation: A Sketch of the H & H Theory.

[54] Perkell J.S. (2012). Movement goals and feedback and feedforward control mechanisms in speech production. J. Neurolinguistics.

[55] Tjaden K., Lam J., Wilding G.E. (2013). Vowel acoustics in Parkinson’s disease and multiple sclerosis: Comparison of clear, loud and slow speaking conditions. J. Speech Lang. Heart Res..

[56] Dromey C., Ramig L.O., Johnson A.B. (1995). Phonatory and articulatory changes associated with increased vocal intensity in Parkinson’s disease: A case study. J. Speech Heart Res..

[57] Sapir S., Spielman J., Ramig L.O., Story B., Fox C. (2007). Effects of intensive voice treatment (the Lee Silverman Voice Treatment (LSVT)) on vowel articulation in dysarthric individuals with idiopathic Parkinson disease: Acoustic and perceptual findings. J. Speech Lang. Heart Res..

[58] Sapir S., Ramig L.O., Spielman J., Fox C. (2010). Formant centralization ratio: A proposal for a new acoustic measure of dysarthric speech. J. Speech Lang. Heart Res..

[59] Baumgartner C.A., Sapir S., Ramig L.O. (2001). Voice quality changes following phonatory-respiratory effort treatment (LSVT) versus respiratory effort treatment for individuals with Parkinson’s disease. J. Voice.

[60] McClean M., Tasko S. (2002). Association of orofacial with laryngeal and respiratory motor output during speech. Exp. Brain Res..

[61] Bryans L.A., Palmer A.D., Anderson S., Schindler J., Graville D.J. (2021). The impact of Lee Silverman Voice Treatment (LSVT LOUD) on voice, communication and participation: Findings from a prospective, longitudinal study. J. Commun. Disord..

[62] Wight S., Miller N. (2015). Lee Silverman Voice Treatment for people with Parkinson’s: Audit of outcomes in a routine clinic. Int. J. Lang. Commun. Disord..

[63] Dykstra A.D., Adams S.G., Jog M. (2015). Examining the relationship beyweem speech intensity and self-rated communicative effectiveness in individuals with Parkinson’s disease and hypophonia. J. Commun. Disord..

[64] Lowit A., Egan A., Hadjivassiliou M. (2020). Feasibility and acceptability of Lee Silverman Voice Treaetment in progressive ataxias. Cerebellum.

[65] Mackenzie C., Lowitt A. (2007). Behavioural intervention effects in dysarthria following stroke: Communication effectiveness, intelligibility and dysarthria impact. Int. J. Lang. Commun. Disord..

