# Supplementary material for: Feasibility of a Telerehabilitation Adaptation of the Be Clear Speech Treatment Program for Non-Progressive Dysarthria
Source: Brain Sci. 2022 Jan 31;12(2):197. doi: 10.3390/brainsci12020197 (PMC8870717; doi:10.3390/brainsci12020197)
Supplement: Supplementary file 1 [file brainsci-12-00197-s001.zip › Figure S1-S_ V 2.pdf]

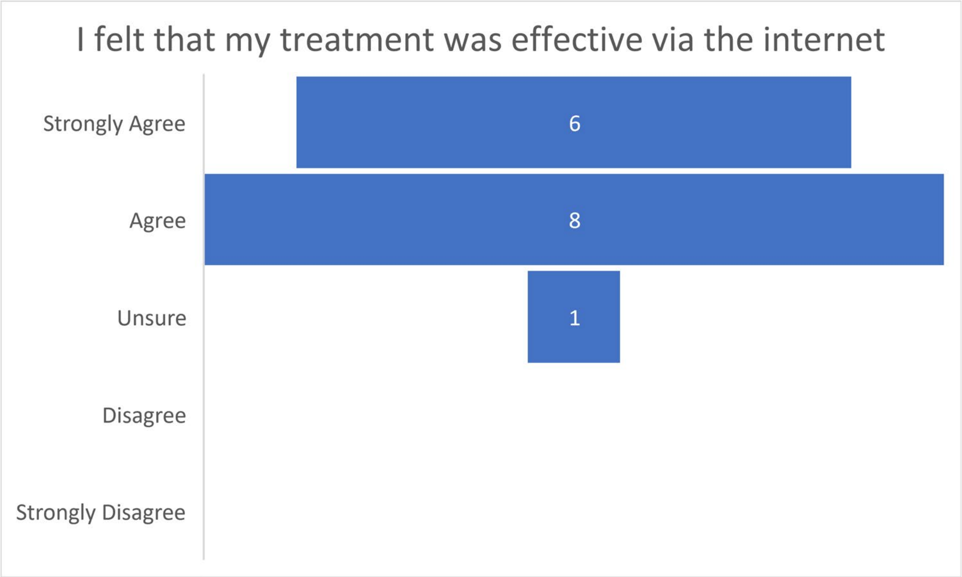

Figure S1. Participant ratings of online treatment effectiveness.

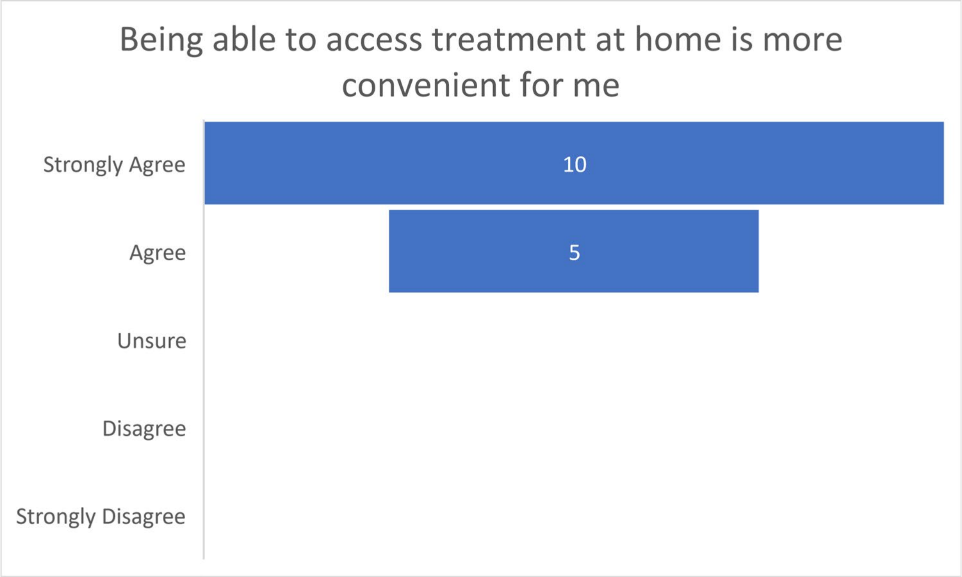

Figure S2. Participant ratings of online treatment convenience.

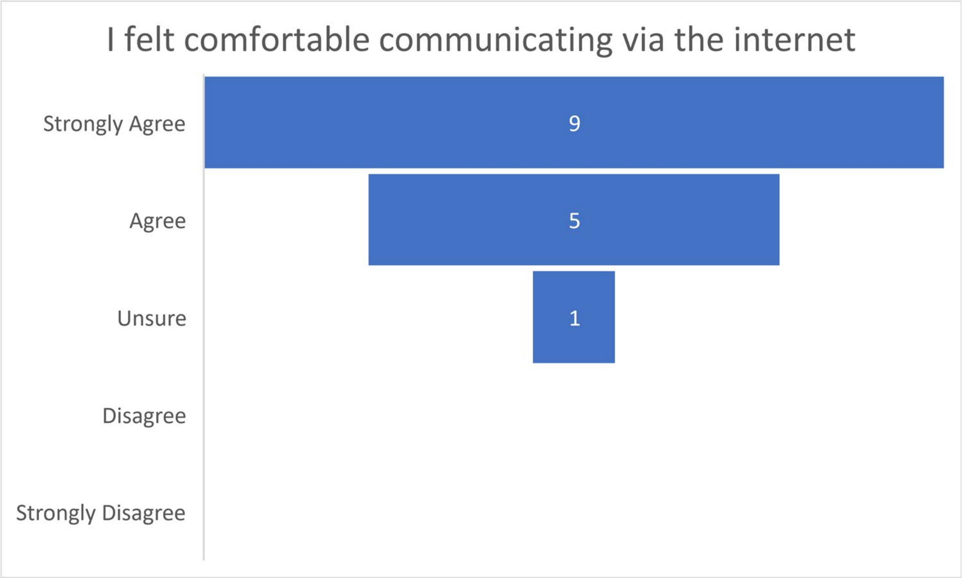

Figure S3. Participant ratings of comfortableness with online treatment.

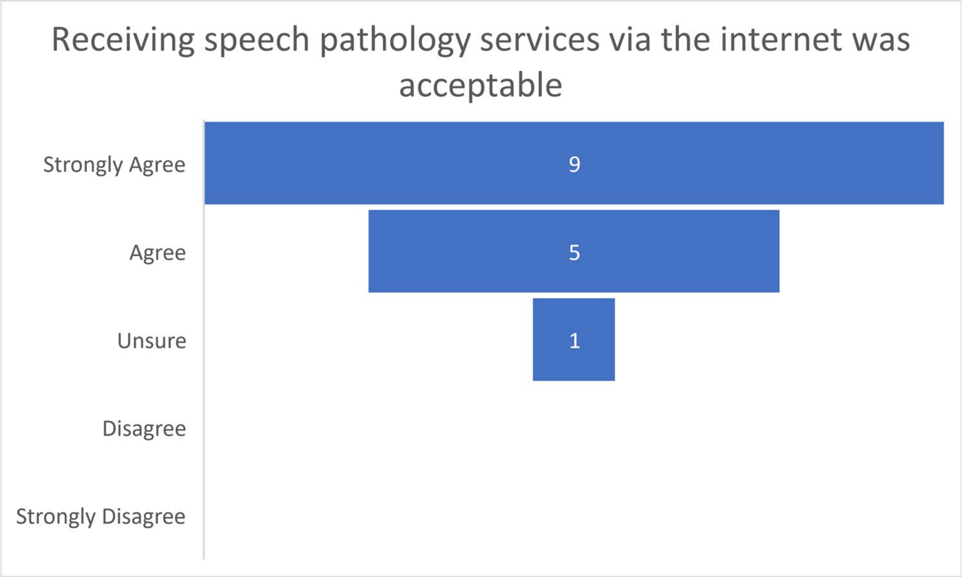

Figure S4. Participant ratings of online treatment acceptability

.

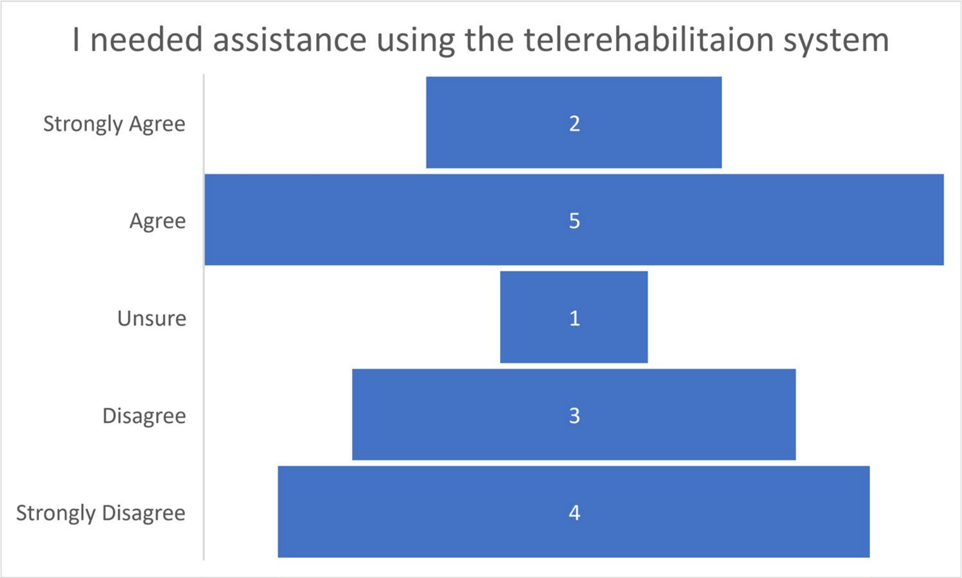

Figure S5. Participant ratings of required telerehabilitation system assistance.

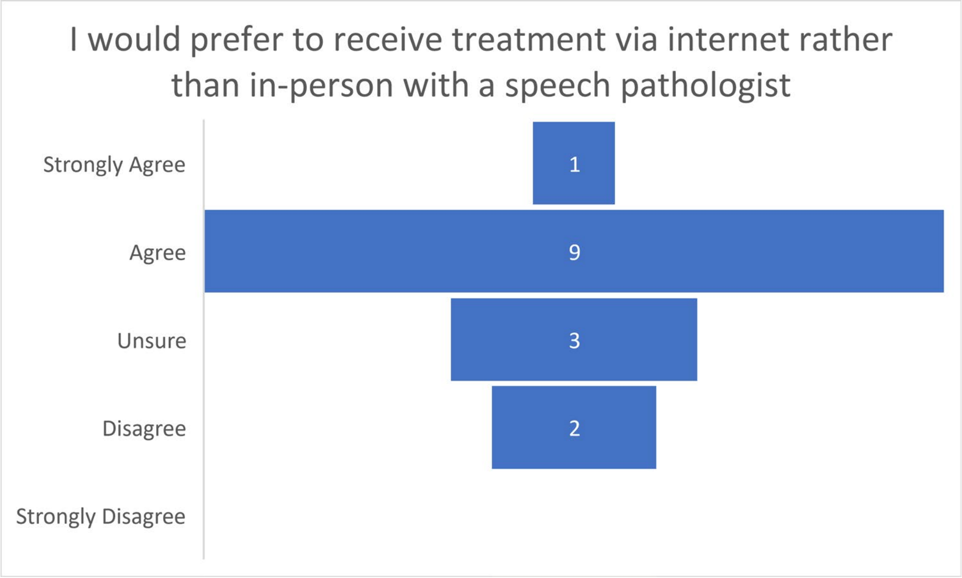

Figure S6. Participant preferences for service delivery**.**
