# Supplementary material for: Feasibility of a Telerehabilitation Adaptation of the Be Clear Speech Treatment Program for Non-Progressive Dysarthria
Source: Brain Sci. 2022 Jan 31;12(2):197. doi: 10.3390/brainsci12020197 (PMC8870717; doi:10.3390/brainsci12020197)
Supplement: Supplementary file 1 [file brainsci-12-00197-s001.zip › Table S1.pdf]

Table S1. Be Clear: OnlineTask analysis

Phase	Components	Task	Interaction	Stimuli	Time	Client Response	Technical Function
Phase 1: Pre-Practice	Extended pre-practice	Shaping and instatement of clear speech concepts	Real-time	Clinician examples of clear and unclear speech, text for reading, audio/ verbal feedback on performance	60 minutes at outset of program	Verbal<br>Rating of clear speech on scale (1-10)	VC, audio recording and playback, display text, white board rating scale
Pre- Phase 2	Correspondence	Determining treatment target saliency	Asynchronous	List of everyday functional phrases & service requests	Unspecified	Written	Emails from Clinician to Client and from Client to Clinician
Phase 2: Intensive Practice	Pre-practice	Shaping and instatement of clear speech concepts using subset of session stimuli	Real time	Random presentation of subset of functional phrases and service requests, reading aloud, picture description and conversation tasks	10 minutes	Verbal rating of clear speech on rating scale (1-10)	VC, display text and picture stimuli, audio recording and playback, whiteboard rating scale
Phase 2: Intensive Practice	Practice	Reading everyday functional phrases and	Real time	Text, and audio/ verbal feedback on	20 minutes	Verbal rating of clear speech on rating	VC, display text, audio recording and

		service requests		performance		scale (1-10)	playback, whiteboard rating scale
Phase 2: Intensive Practice	Practice	Functional speech tasks: Reading aloud; Picture description; Conversation	Real time	Text, picture stimuli and audio/ verbal feedback on performance	30 minutes	Verbal rating of clear speech on rating scale (1-10)	VC, display text and picture stimuli, audio recording and playback, whiteboard rating scale
Phase 2: Independent practice	Home Practice	Functional phrases & service requests, functional speech tasks, and transfer task	Offline	Text	15 minutes	Verbal	USB drive, recording device such as phone

Note: VC = videoconferencing
