# Supplementary material for: Feasibility of a Telerehabilitation Adaptation of the Be Clear Speech Treatment Program for Non-Progressive Dysarthria
Source: Brain Sci. 2022 Jan 31;12(2):197. doi: 10.3390/brainsci12020197 (PMC8870717; doi:10.3390/brainsci12020197)
Supplement: Supplementary file 1 [file brainsci-12-00197-s001.zip › Table S2.pdf]

Table S2. Intra-rater reliability intraclass correlations (ICC) for 10 naïve listeners

**Rater**	**ICC**	**95% Confidence Interval**		**F**	**p**
		Lower bound	Upper bound		
1	.699	.051	.911	3.55	.023
2	.879	.582	.965	7.85	.001
3	.939	.796	.982	16.27	.0001
4	.814	.324	.947	5.00	.006
5	.304	-1.748	.806	1.41	.290
6	.294	-1.287	.792	1.42	.283
7	.912	.704	.974	12.40	.0001
8	.686	-.124	.910	3.05	.038
9	.792	.329	.939	5.20	.005
10	.807	.299	.945	4.83	.007
